# p14ARF Post-Transcriptional Regulation of Nuclear Cyclin D1 in MCF-7 Breast Cancer Cells: Discrimination between a Good and Bad Prognosis?

**DOI:** 10.1371/journal.pone.0042246

**Published:** 2012-07-30

**Authors:** Eileen M. McGowan, Nham Tran, Nikki Alling, Daniel Yagoub, Lisa M. Sedger, Rosetta Martiniello-Wilks

**Affiliations:** 1 Translational Cancer Research Group, School of Medical and Molecular Biosciences, Faculty of Science and Centre for Health Technologies, University of Technology Sydney, Sydney, New South Wales, Australia; 2 Sydney Medical School, The University of Sydney, Sydney, New South Wales, Australia; 3 Flow Cytometry Unit, Garvan Institute of Medical Research, Sydney, New South Wales, Australia; 4 School of Biotechnology and Biomolecular Sciences, University of New South Wales, Sydney, New South Wales, Australia; Virginia Commonwealth University, United States of America

## Abstract

As part of a cell’s inherent protection against carcinogenesis, p14ARF is upregulated in response to hyperproliferative signalling to induce cell cycle arrest. This property makes p14ARF a leading candidate for cancer therapy. This study explores the consequences of reactivating p14ARF in breast cancer and the potential of targeting p14ARF in breast cancer treatment. Our results show that activation of the p14ARF-p53-p21-Rb pathway in the estrogen sensitive MCF-7 breast cancer cells induces many hallmarks of senescence including a large flat cell morphology, multinucleation, senescence-associated-β-gal staining, and rapid G1 and G2/M phase cell cycle arrest. P14ARF also induces the expression of the proto-oncogene cyclin D1, which is most often associated with a transition from G1-S phase and is highly expressed in breast cancers with poor clinical prognosis. In this study, siRNA knockdown of cyclin D1, p21 and p53 show p21 plays a pivotal role in the maintenance of high cyclin D1 expression, cell cycle and growth arrest post-p14ARF induction. High p53 and p14ARF expression and low p21/cyclin D1 did not cause cell-cycle arrest. Knockdown of cyclin D1 stops proliferation but does not reverse senescence-associated cell growth. Furthermore, cyclin D1 accumulation in the nucleus post-p14ARF activation correlated with a rapid loss of nucleolar Ki-67 protein and inhibition of DNA synthesis. Latent effects of the p14ARF-induced cellular processes resulting from high nuclear cyclin D1 accumulation included a redistribution of Ki-67 into the nucleoli, aberrant nuclear growth (multinucleation), and cell proliferation. Lastly, downregulation of cyclin D1 through inhibition of ER abrogated latent recurrence. The mediation of these latent effects by continuous expression of p14ARF further suggests a novel mechanism whereby dysregulation of cyclin D1 could have a double-edged effect. Our results suggest that p14ARF induced-senescence is related to late-onset breast cancer in estrogen responsive breast cancers and/or the recurrence of more aggressive breast cancer post-therapy.

## Introduction

The tumour suppressor gene p53 is central to determining whether cells enter differentiation, apoptosis or senescence [1], [2], [3], [4], [5], [6]. Among the many upstream genes that regulate p53, the tumour suppressor p14ARF, encoded by the *CDKN2A* locus, initiates the p53/p21^WAP/CIP^ pathway to suppress abnormal cell proliferation in response to hyperproliferative oncogenic signals [7], [8], [9], [10]. Activation of the *CDKN2A* locus by oncogenes, as a protective mechanism against carcinogenesis, is well reported [8], [9], [11]. Consistent with its protective role, this locus is frequently deleted in human cancers, resulting in p14ARF loss-of-function. In breast cancer p14ARF is often deleted, mutated or inappropriately methylated [12], [13]. The physical association of p14ARF with hdm2 prevents the ubiquitylation and subsequent degradation of p53 [7], [8], [14], [15], thus stabilising and increasing p53 protein levels. P53 is then responsible for the transcription of p21 and other critical cell-cycle regulatory genes. Clinically, p14ARF is a good candidate target for senescence-type cancer therapies due to its intrinsic cell cycle inhibitory properties. Mimics, or the use of chemical inhibitors of p14ARF function such as Nutlin-3, which targets human double minute [hdm2] protein have received considerable attention as potential cancer treatments [16], [17], [18], [19].

The proto-oncogene cyclin D1 is overexpressed in response to estrogen activation in human estrogen receptor (ERα) positive breast cancers and is associated with the mitogenic effects of ERα and onset of carcinogenesis [20]. Cyclin D1 repression by either p19ARF (p14ARF mouse homolog), or p53 or p21 has been suggested to contribute to the tumour suppressor function of these genes [21], [22], [23]. Furthermore, p21 through initiation of the p19ARF pathway has been shown to inhibit cyclin D1-cdk activities [24]. In addition, overexpression of p21 (downstream of p53) has been shown to selectively control the activity of ERα, inducing a senscence-like phenotype [22] and therefore providing protection against carcinogenesis. In contrast, others have shown that cyclin D1 is upregulated when the p53 pathway is initiated [25], [26].

Accelerated senescence and chemotherapeutic agents enriching for a senescence-phenotype in cancer have been heavily advocated in cancer therapy [27], [28], [29], [30], [31]. In order to use accelerated senescence as a safe therapeutic, it is important to understand the consequences of reactivating this pathway in breast cancer. Our previous studies have shown that p14ARF induction in MCF-7 epithelial breast cancer cells leads to of the development of highly metabolically active senescent-like cells, that potentially contribute to latent recurrence or irreversible inhibition of cell proliferation post-p14ARF treatment [32]. Here we describe a novel role for p14ARF in the post-translational regulation of cyclin D1 and its inter-relationship with p53 and p21. We showed p21 is pivotal in maintaining high cyclin D1 expression and cell-cycle inhibition; cells maintaining high levels of p14ARF and p53 and low p21 continue to proliferate. Importantly, we are the first to describe that p14ARF regulation of cyclin D1 and Ki-67 localisation is contributory to both rapid inhibition of DNA synthesis and cell cycle arrest, and causally associated with abnormal nuclear proliferation in latent breast cancer cells. Furthermore, the pure anti-estrogen, ICI 182780, blocks latent cell cycle progression implicating ERα as an accessory in repressing the senescent phenotype.

## Results

### Characterisation of p14ARF Effects on MCF-7 Cell Cycle

**Figure 1:**
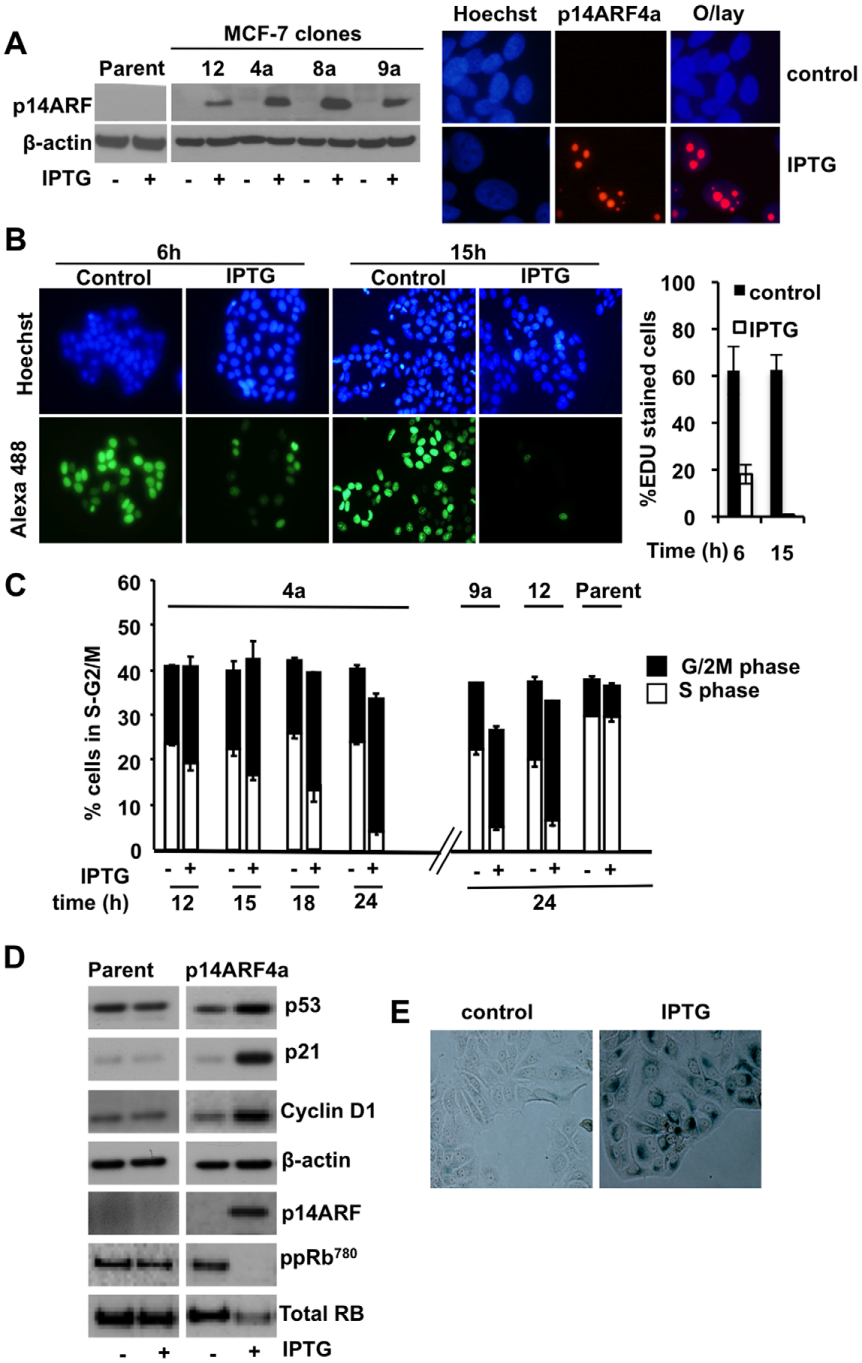
P14ARF reactivates the p53 pathway and increases cyclin D1 protein in MCF-7 cells. **A.** MCF-7p14ARF clonal cell lines (12, 4a, 8a and 9a) were induced with IPTG and levels of p14ARF were analysed by Western blot. β-actin was used as a loading control. The subcellular distribution of p14ARF (red staining) was analysed by immunofluorescence staining. Nuclei were stained with Hoechst 33342 (blue). **B.** Cells were grown on cover slips in 6-well plates and EDU was added directly to the cell culture medium 6h and 15h post-IPTG (5 mM) treatment and cells were incubated for a further 20h. Images of individual cells undergoing DNA synthesis within this 20h period were captured. Proliferating cells were visualised using Alexa 488 (green) and nuclei using Hoechst (blue). Column graph: % EDU stained cells (+/− SD). **C.** MCF-7p14ARF, MCF-7 parent +/− IPTG, were harvested over a time period of 24h and cell cycle phases analysed by flow cytometry. **D.** 24h +/− IPTG, sample proteins were analysed by Western blot. Relative expression of p53, p21, cyclin D1, total Rb and pRb phosphorylated at Ser^780^ [pRb (Ser^780^)], and p14ARF are shown. **E.** MCF-7p14ARF, clone 4a cells were treated +/− IPTG for 5 days and stained with SA-β-gal to detect senescence.

We engineered MCF-7p14ARF cells which inducibly express p14ARF in response to IPTG treatment (Figure 1A). To determine the effects of p14ARF on cell proliferation we examined 1) newly synthesised DNA post-p14ARF using EDU chemistry and 2) cell cycle arrest using flow cytometry. DNA synthesis was inhibited in the majority of cells 15h post-p14ARF induction (>80% at 6h and >99% at 15h) shown by lack of EDU incorporation (Figure 1B). Flow cytometric analysis showed less than 2% of cells were present in the early S-phase of the cell cycle at 15h in the p14ARF induced cells (Figure S1A) compared with uninduced and parent cell lines (Figure S1B and C). This finding is consistent with early inhibition of DNA synthesis (Figure 1B) and reports showing genes involved in DNA replication are highest during early S-phase [33]. Cells progressed through mid, late S-phase and accumulated in G2/M phase of the cell cycle at 24h (Figure 1C and Figure S1A). Little variation in cell cycle phase distribution was observed in the untreated (control) cells and the parent MCF-7 cells (+/− IPTG) (Figure S1B and C). To confirm this finding was not a result of clonal aberration, we repeated the same experiments in 2 other clones (9a and 12). Clones 4a, 9a and 12 showed a similar pattern of cell cycle arrest with accumulation of cells in the G2/M phase at 24h (Figure 1C). In comparative experiments we used the pure anti-estrogen ICI 182780 (known to induce quiescence) and showed MCF-7p14ARF cells arrested in the G0/G1 phase of the cell cycle [32], which is consistent with other reports [34].

### P14ARF Reactivates the p53 Pathway and Increases Cyclin D1 Protein in MCF-7 Cells

Induction of p14ARF upregulated the protein expression of p53 and p21; dephosphorylated and downregulated the retinoblastoma protein (Rb); and surprisingly increased cyclin D1 protein expression (Figure 1D). MCF-7 cells treated with IPTG expressing p14ARF were positive for the senescence marker senescence-associated-β-galacotosidase (SA-β-Gal), which was absent in the untreated cells (control) (Figure 1E – represented by clone 4a). Induction of p14ARF in all the MCF-7p14ARF clones tested showed similar characteristics: rapid cell cycle arrest, accumulation of cells in G2/M, reactivation of the p53-p21 pathway, increased cyclin D1 expression, and expression of the senescence marker SA-β-Gal. To determine if the effects of p14ARF in cell cycle and cell growth were reversible we induced p14ARF for 4h –24h with IPTG (Figure S2A) and removed the IPTG by replacing the medium with IPTG-free medium for a further 3 days. Cell cycle (Figure S2B) and cell size (Figure S2C) were analysed. Continuous expression of p14ARF protein increased the cells commitment to cell cycle arrest in G2/M phase and increased cell growth and reduced the likelihood of cells re-entering the cell cycle.

### Post-transcriptional Regulation of Cyclin D1 by p14ARF in MCF-7 Cells

Seemingly conflicting reports have shown that cyclin D1 is downregulated by p19ARF (mouse homolog of p14ARF) [23] and p53 [21], [22] in MCF-7 cells and other reports have shown that cyclin D1 is upregulated when this pathway is initiated [25], [26]. Our study is consistent with the later observation. The following experiments were performed using the MCF-7p14ARF-4a cells, however key findings were verified using different clones and the results were found to be consistent.

### Quantitative Analysis of mRNA and Protein Expression of Cyclin D1, p53, and p21 by p14ARF

**Figure 2:**
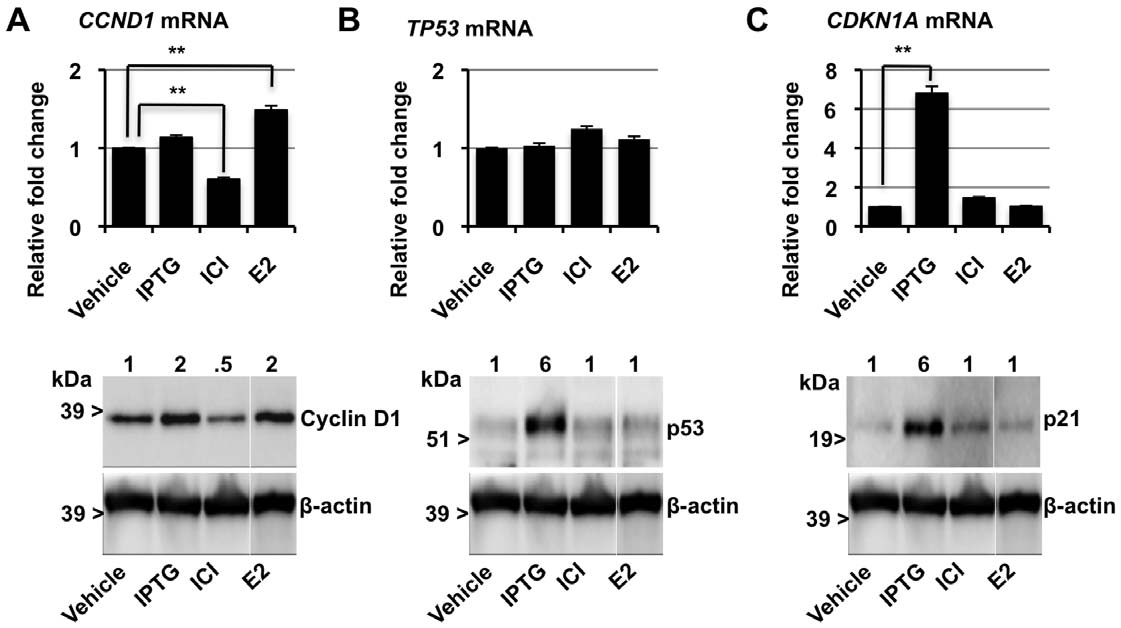
Regulation of *cyclin D1, p53, and p21* mRNA and protein expression of in MCF-7p14ARF. Cells were treated with IPTG, ICI 182780 (ICI), or E2 for 12 h. Top panel: relative gene expression of **A.** *CCDN1*, **B.** *TP53* and **C.** *CDKN1A* mRNA quantified by qRT-PCR and relative expression normalised to control *ACTB. Columns,* Data represents the mean ± SEM of three separate experiments (** P<0.001. number = 9). Bottom panel: representative Western blots of **A.** cyclin D1, **B.** p53, and **C.** p21. Relative levels of protein are shown above each lane. β-actin was used as a control. The representative images are all from the same sample set, run on the same gel and Western blot.

Cyclin D1 is an important mitogen in breast cancers and is associated with poor prognosis in breast cancer patients [35]. To gain further insight into p14ARF upregulation of cyclin D1 we analysed the expression of cyclin D1 *(CCDN1)* at the mRNA level using quantitative RT-PCR (qRT-PCR) and analysed the protein expression by western blot post-IPTG induction of p14ARF (Figure 2 panel A). For controls we used the pure anti-estrogen ICI 182780, which is a well-characterised inhibitor of cyclin D1, and secondly estrogen - a known stimulant of cyclin D1. These results were compared to vehicle alone. Our results showed that induction of p14ARF with IPTG-treatment for 12h had no significant effect on *CCDN1* mRNA levels, however cyclin D1 protein was increased >2-fold (Figure 2A). The discrepancy between the cyclin D1 RNA expression and protein levels post-p14ARF induction suggests a post-transcriptional mechanism is responsible. In contrast and as expected ICI 182780 decreased *CCDN1* mRNA and cyclin D1 protein approximately 2-fold (Figure 2A). Estrogen increased *CCDN1* mRNA and cyclin D1 protein (Figure 2A).

In addition, we performed a comparative analysis of *TP53* and *CDKN1A* mRNA and p53 and p21 protein levels in the same samples (Figure 2B and C respectively). Direct comparison of p53 mRNA levels showed no increase in *TP53* mRNA transcription and conversely p53 protein significantly increased with IPTG-treatment (>6 fold). IC1 182780- and estrogen- treatments had little effect on the expression of p53 at both the mRNA and protein levels in the same experiment. Increased expression of both *CDKN1A* mRNA (>6 fold) and p21 protein (>6 fold) was observed with IPTG treatment, whereas no significant change was shown post-ICI 182780 and estrogen treatments (Figure 2C).

In summary, the change we observed with p53 and p21 at the transcription and translational level were consistent with previous reports, and the disparity between p14ARF regulation of cyclin D1 protein and *CCDN1* mRNA merited further investigation.

### Post-transcriptional Regulation of Cyclin D1 by p14ARF

**Figure 3:**
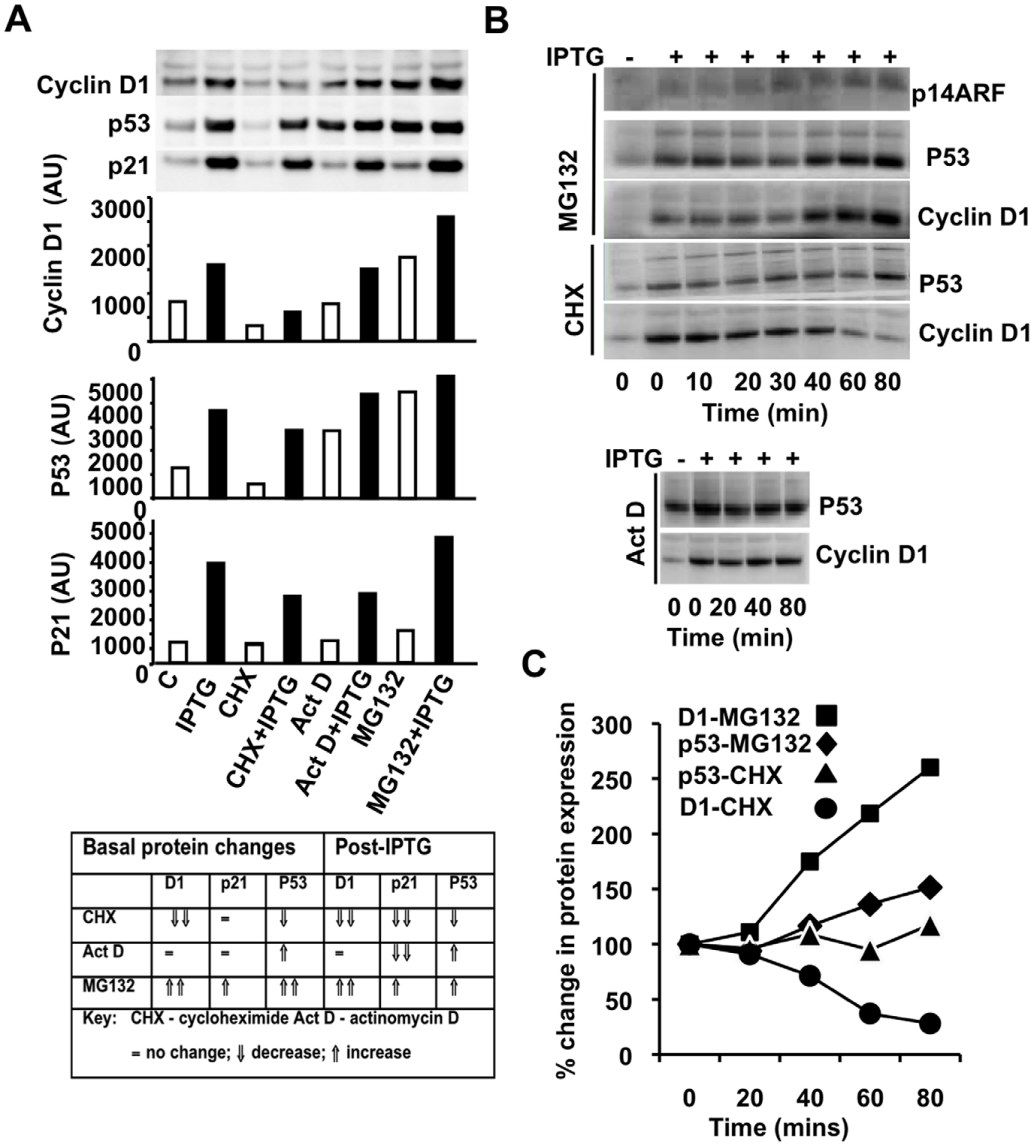
Comparative analysis of p14ARF transcriptional and post-transcriptional regulation of cyclin D1, p53 and p21 by p14ARF. MCF-7p14ARF 4a cells were treated with IPTG (lanes, 2, 4, 5, 8) or control (lanes 1, 3, 5, 7). Cells were subsequently treated with CHX, Act D, MG132 or vehicle (control). **A.** Cyclin D1, p21 and p53 protein expression was analysed by Western blot, post-treatments as indicated. *Column graph:* Protein expression was analysed by densitometry and expressed as arbitrary units (AU). **B.** Time course of p53, cyclin D1 expression in IPTG induced MCF-7p14ARF cells with MG132, CHX and Act D. Representative Western blot from three experiments show relative protein expression of p14ARF, p53 and cyclin D1. C. Line graph of % change in cyclin D1 and p53 protein expression when compared to control post MG132 and CHX treatments.

MCF-7 p14ARF cells were induced with p14ARF for 24h and subsequently treated with the transcriptional inhibitor, actinomycin D (Act D), and the translational protein synthesis inhibitor, cycloheximide (CHX), for a further 60 mins. The treatment timing of CHX was based on the cyclin D1 turnover rate of approximately 30 min [36]. CHX treatment decreased basal levels of cyclin D1 (Figure 3) and completely blocked any increase in cyclin D1 protein expression post-IPTG treatment (Figure 3). Any increase in cyclin D1 expression by p14ARF in the 24h preceding CHX treatment was also abrogated (levels of cyclin D1 post-IPTG-CHX treatment were similar or below control treated cells; Figure 3). Addition of Act D had no effect on cyclin D1 expression in untreated (Fig 3, compare lanes C and Act D) and IPTG-treated cells (Figure 3, compare lanes IPTG and Act D+ IPTG), supporting our previous findings that cyclin D1 was not regulated by p14ARF at the transcriptional level. We also used MG132, a proteasome inhibitor, to determine whether protein turnover was impeded by p14ARF induction. Treatment with MG132 increased cyclin D1 expression in the presence and absence of IPTG (Figure 3, compare C and MG132, IPTG and MG132+ IPTG, respectively) supporting cyclin D1 protein post-translational regulation by p14ARF.

We also analysed the effects of CHX and Act D on p53 and p21 protein expression in MCF-7p14ARF cells +/− IPTG. IPTG induction of p14ARF increased protein expression of p53 and p21 (Figure 3, compare C and IPTG). Levels of P53 protein increased with Act D treatment in the presence or absence of p14ARF, consistent with one of the earliest observations of this pathway [37] whereas p21 expression levels decreased (Figure 3, compare lanes Act D+/− IPTG). A decrease in p21 protein expression was observed with CHX treatment (Figure 3, compare lanes CHX + IPTG and IPTG). Treatment with MG132 increased protein expression of both p53 and p21 (Figure 3, compare lanes MG132+ IPTG and IPTG). These results are consistent with known regulatory pathways of p14ARF-p53-p21 whereby p14ARF sequesters the ubiquitinase hdm2, allowing stabilisation of p53 protein. Basal expression of cyclin D1 and p53 increasing 2 and 4-fold respectively in MG132 treated cells (Figure 3, compare C and MG132) consistent with a dependence on proteasome regulation of these proteins. Comparative analysis of protein expression is shown in Figure 3A). These studies were repeated and showed the same pattern of protein expression.

To further examine the influence of p14ARF on cyclin D1 turnover we treated MCF-7 cells with IPTG for 24h and then treated the cells with MG132, CHX and Act D over a period of 10 to 80 min (Figure 3B and C). Cyclin D1 increased approximately 2-fold within 50 min of MG132 treatment and decreased 2-fold with CHX treatment whereas Act D had no effect on cyclin D1 expression (Figure 3B and C). Comparatively MG132 increased p53 by increased <50% of control at 50 min whereas CHX and Act D had little or no effect on p53 expression (Figure 3B). Expression of p14ARF is very low in these cells however we did observe a slight increase in p14ARF expression over time with MG132 (Figure 3B) but no apparent changes with CHX and Act D (data not shown).

### p21 is Required for Cyclin D1 Stable Expression and Cell Cycle Inhibition Post-p14ARF Induction

**Figure 4:**
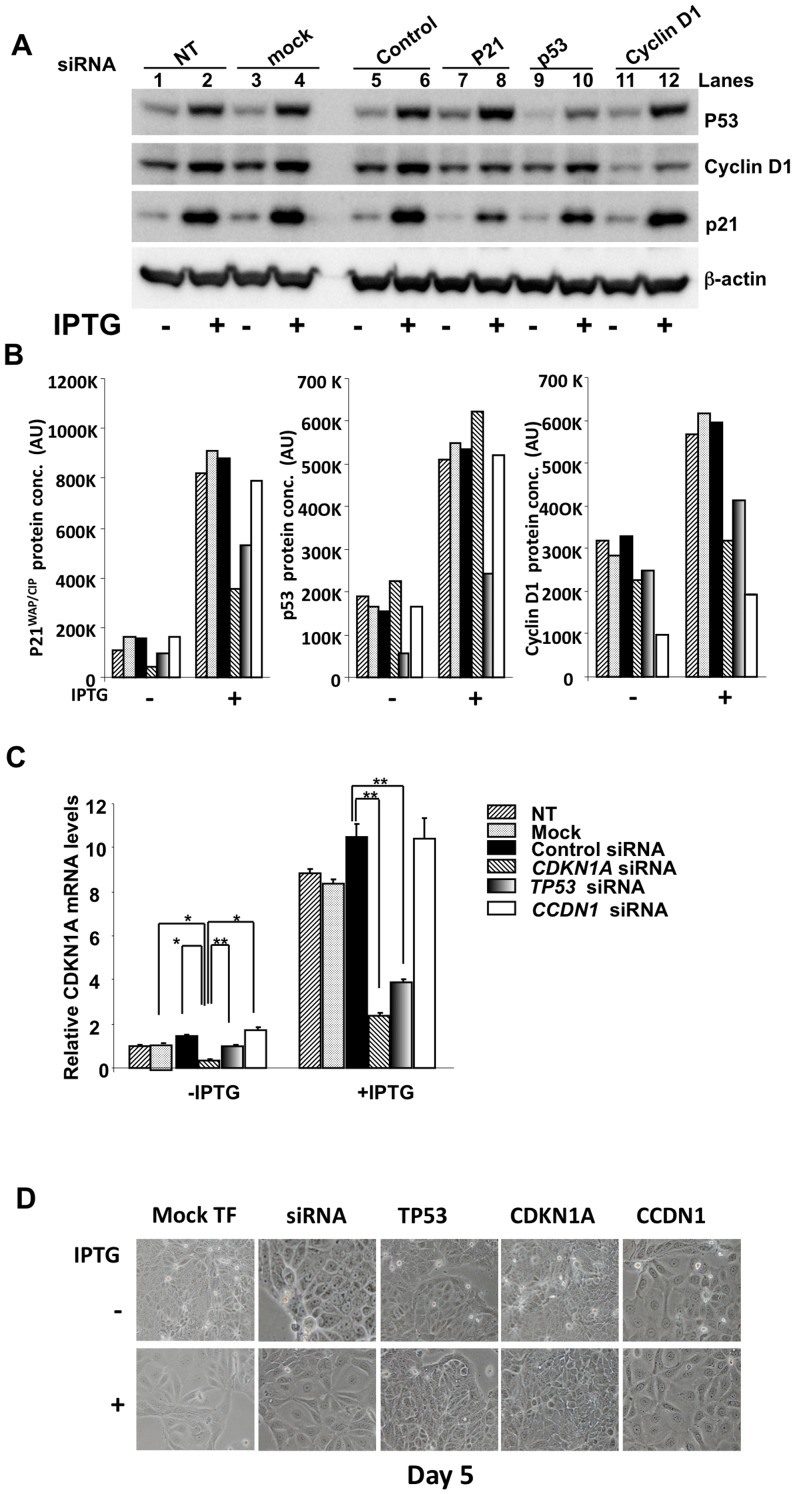
p21 is required for cyclin D1 stable expression post-p14ARF induction and cell inhibition. siRNA knockdown of *CDKN1A*, *TP53* and *CCND1* in MCF-7 ^p14ARF^ cells. For siRNA knockdown (KD) experiments, cells were seeded at 2.0×10^5^ cells for 18h. Cells were transfected with siRNA complexes, *CCND1 (cyclin D1), TP53 (p53), and CDKN1A (*p21*)*, and negative control siRNA. No treatment and mock transfection were included for comparative analysis. BLOCK-iT™ Fluorescent Oligo was co-transfected as an RNA transfection control. Twenty-four hours following transfection, cells were stimulated with or without IPTG (5mM) for a further 24h and then protein was extracted for Western blot analysis. **A.** Representative Western blots of relative p53, cyclin D1 and p21 protein expression. **B.** Protein expression of p53, cyclin D1, and p21, was measured for siRNA transfection using Kodak IM software and expressed as arbitrary units (AU). **C.** Comparative qRT-PCR analysis of CDKN1A, post -*CCDN1*, -*TP53* and –*CDKNA1* siRNA knockdown. Experiments were performed in technical triplicate on three separate occasions (*P>0.001, N = 9). **D.** Representative live cell images 5 days post-IPTG induction, following 24h siRNA transfection (magnification ×200).

Given the conflicting literature with our findings on the regulation of cyclin D1 by p53 and p21 [21], [22], [25] (Figure 1 and 2), we conducted a systematic siRNA knockdown (KD) of *CDKN1A* (p21), *TP53* (p53), and *CCND1* (cyclin D1), to understand their interactive relationship. We achieved a 65–80% decrease in basal protein expression upon siRNA transfection when analysed at 18h, and this was confirmed by co-transfection with a control fluorescent oligo (Invitrogen) and Western blot analysis (data not shown). Induction of p14ARF in MCF-7 after KD of p21 (*CDKN1A*) resulted in decreased cyclin D1 expression (similar to control) with no reduction in p53 expression (Figure 4A compare lanes 2,4,6 with lane 8); KD of p53 (*TP53*) decreased expression of p21 and cyclin D1 (Figure 4A, compare 2, lanes 2,4,6 with lane 10). Previously it has been shown that cyclin D1 is necessary for p21 stability [38], however here we showed that KD of cyclin D1 with *CCDN1 siRNA* had no effect on p53 and p21 protein expression (Figure 4A, compare lanes 2,4,6 with lane 12). Quantitative comparative analysis of protein expression is represented in Figure 4B. In addition, KD of cyclin D1 using *CCDN1* siRNA had no effect on *CDKN1A* mRNA, whereas reciprocal experiments with *TP53* significantly decreased *CDKN1A* mRNA (Figure 4C). Images of the cells 5 days post siRNA transfections +/− IPTG, showed that p53 and p21 were necessary for the anti-proliferative and increased cell growth induced by p14ARF, whereas depletion of cyclin D1+/− IPTG was not (Figure 4D). Table 1 summarises the protein changes post-IPTG induction after systematic siRNA knockdown of *CDKN1A* (p21), *TP53* (p53), and *CCND1* (cyclin D1).

**Table 1:** 

Protein	*CCDN1*Knockdown	*CDKN1A*Knockdown	*TP53*Knockdown
D1	⇓	⇓⇓	⇓
P21	=	⇓⇓	⇓⇓
P53	=	⇑	⇓⇓

### Cyclin D1 is not Required for Increase in Cell Growth

**Figure 5:**
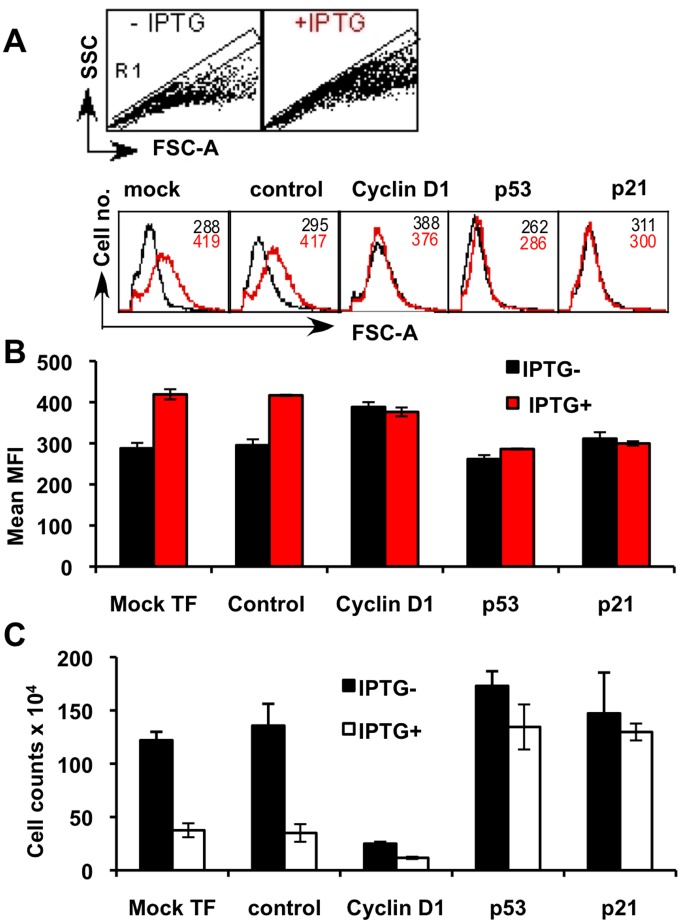
Cyclin D1 is not required for p14ARF-induced cell growth (size, [forward scatter; FSC-A]). MCF7 cells were transfected in triplicate with siRNA as described in Figure 4, and cells were harvested at day 3 and analysed using a BD LSRII cytometer. Flow cytometry data (20,000 events) were acquired using DIVA software (version 6.1.3), analysed with CellQuest Pro (version 6) and composite figures were generated with CANVAS (version X) software. **A.** To eliminate cell doublets and clumps from the analysis, single cells were gated (R1) from FSC-A versus FCS-H dot plots. A representative plot only is shown from mock-transfected cells. FSC-A histograms of the R1 populations were then used to indicate relative cell size of MCF-7 cells ± IPTG treatment after siRNA knockdown of cyclin D1, p53 and p21. Black line shows cells without IPTG and the red line shows cells treated with IPTG. **B.** Mean Fluorescent Intensity (MFI) analysis of FSC-A from triplicate biological experiments; data shown are mean MFI ± SD. **C.** Cell counts were performed using a haemocytometer. Data were presented as mean cell counts (x10^4^) ± SD performed on three separate occasions.

To confirm that cyclin D1 was not required for p14ARF-induced cell growth we measured cell size +/− IPTG post- siRNA knockdown (KD) of *CCDN1* and directly compared these results with *TP53* and *CDKN1A* siRNA KD using Flow Cytometry (Figure 5A). Depletion of cyclin D1, −/+ IPTG, showed that cyclin D1 was not required for increased cell size (Figure 5A, B), as expected cyclin D1 was essential for cell cycle progression (Figure 5C). siRNA knockdown of p53 and p21 followed by +/− IPTG treatment resulted in an abrogation of cell growth (Figure 5A and B) and cell cycle arrest (Figure 5C). Importantly, cells expressing high p53 and low cyclin D1, where p21 had been depleted using siRNA-*CDKN1A*, continued to proliferate (Figure 5C). Comparative analysis of these data strongly suggested that p21 was pivotal in cell cycle regulation in these experiments. Low p21 expression, whether it was through knockdown with *CDKN1A* or *TP53* siRNAs resulted in proliferation in the presence or absence of p53 protein. Conversely, high p21 expression correlated with cell-cycle inhibition in the presence or absence of cyclin D1. These results were performed in technical triplicates in duplicate biological experiments with the same results, which are summarised in Table 2.

**Table 2:** 

Control	*CCDN1*Knockdown	*CDKN1A*Knockdown	*TP53*Knockdown
DI-highp21-highP53-high** = Inhibition**	D1-lowp21-highp53-high** = Inhibition**	D1-lowp21-lowP53-high** = Proliferation**	D1-lowp21-lowp53-low** = Proliferation**

### High Nuclear Cyclin D1 Expression Inversely Correlates with Ki-67 Post-p14ARF Cell Cycle –Arrest

**Figure 6:**
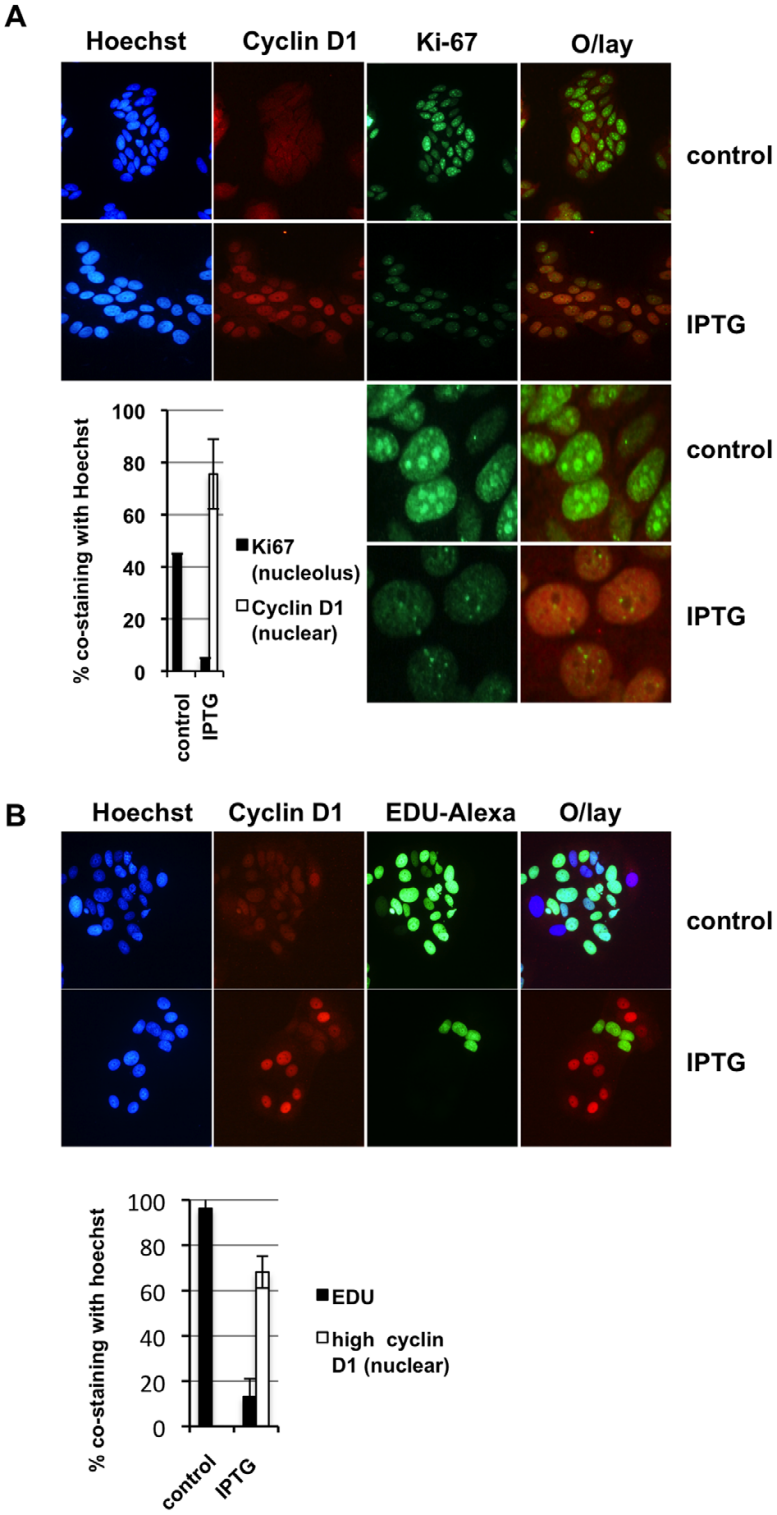
High nuclear cyclin D1 expression inversely correlates with Ki-67 and EDU proliferation markers post-p14ARF cell cycle –arrest. **A.** MCF-7 cells were seeded on cover slips for 48h and subsequently treated with 5 mM IPTG for a further 48h. **A.** Cells were fixed and immunofluorescence stained for cyclin D1, Ki-67, and Hoechst 33342. *Column graph inset:* Over 400 cells per treatment were counted for nuclear cyclin D1 (red staining) (white bar) and nucleolar Ki-67 (green staining) (black bar) and represented as a percentage of total nuclei (blue staining) (+/− SD). **B. EdU incorporation post IPTG (p14ARF) treatment.** EdU was added to the medium of live cells 48h post-IPTG treatment and incubated for a further 20h. EdU incorporation was visualised by staining with Alexafluor 488 (green). The nucleus was stained with Hoechst 33342 (blue) and images were taken on a Nikon Ti Eclipse fluorescence microscope (magnification x200). *Column graph inset:* % cells staining for EdU compared to Hoechst 33342 stained nuclei (+/− IPTG). A minimum of 400 cells was counted for each treatment. These experiments were performed in duplicate on two separate occasions.

The next logical step was to determine the location of cyclin D1 post-p14ARF and compare cyclin D1 expression with markers of proliferation. As shown in Figure 6A, in the control cells cyclin D1 was evenly distributed in the nuclear and cytoplasmic compartments, whereas Ki-67 was highly expressed in the nucleus. In over 40% of the cells, Ki-67 was concentrated in the nucleolus (Figure 6A inset). In contrast, IPTG-treatment resulted in cyclin D1 preferential accumulation in the nucleus, which inversely correlated with Ki-67 expression and notable loss of Ki-67 in the nucleolus (Figure 6A and inset). We further analysed the expression of cyclin D1 expression using EdU Click-iT live cell culture proliferation assays. In these experiments cells were treated with IPTG or vehicle (control) for 48h and EDU was added for a further 20h to capture the majority of proliferating cells. Post-IPTG treatment cyclin D1 accumulated in the nucleus of non-proliferating cells (Figure 6B). Analysis of a minimum of 400 cells per treatment showed a correlation between high nuclear cyclin D1 and inhibition of DNA synthesis (Figure 6B inset). In summary, accumulation of nuclear cyclin D1 was associated with cell-cycle arrest, and nuclear cyclin D1 emergence was associated with proliferation as shown by 2 independent methodologies.

### Abnormal Nuclear Growth and all Proliferation is Associated with Co-expression of Cyclin D1 and Ki-67 in the Nucleolus of Latent p14ARF Cell Cycle Arrested Cells

**Figure 7:**
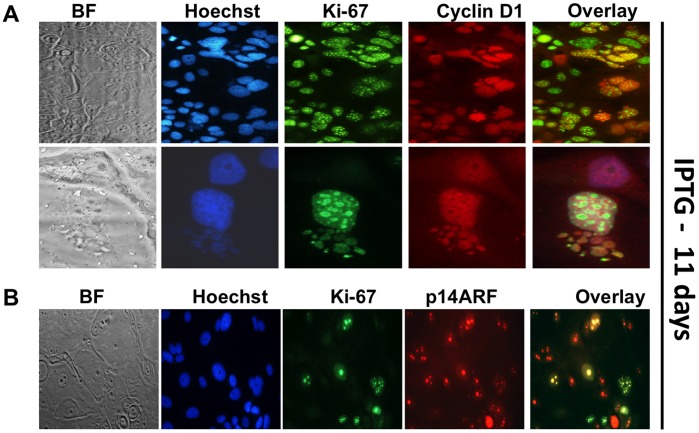
Abnormal nuclear growth and proliferation is associated with co-expression of cyclin D1 and Ki-67 in the nucleolus of latent p14ARF cell cycle arrested cells. MCF-7 cells were seeded on cover slips for 48h and subsequently treated with 5 mM IPTG for a further 11 days. **A.** Cells were fixed and immunofluorescent stained for cyclin D1 (red staining), Ki-67 (green staining), and Hoechst 33342 (blue staining). **B.** Cells were stained for p14ARF (red staining). Images were taken on a Nikon fluorescence microscope (magnification x200).

To determine a requirement for cyclin D1 in p14ARF-induced senescence we monitored MCF-7 cells post IPTG-treatment for a minimum of 2 weeks. In our experiments we consistently observed a fraction of cells exit their mitotic cycle in favour of endoreduplication with an accumulation of multinucleated cells showing elevated nuclear gene content and polyploidy (Figure S3). We co-stained these latent cells with cyclin D1 and Ki-67. Cells treated with IPTG for 11 days showed high levels of nuclear cyclin D1 and high levels of nuclear Ki-67 (Figure 7A). High accumulation of Ki-67 was observed in the nucleolus (Figure 7A). Nuclear budding and abnormal nuclear growth, as visualised by Hoechst 33342 staining, was associated with accumulation of cyclin D1 and Ki-67 (Figure 7A). Ki-67 and p14ARF co-localised in the nucleolus in latent multinucleated cells (Figure 7B), suggesting that loss of p14ARF expression was not the exclusive cause of cell proliferation recurrence in these latent breast cells.

### Cell Cycle Recurrence is Abrogated by Anti-estrogen ICI 182780

**Figure 8:**
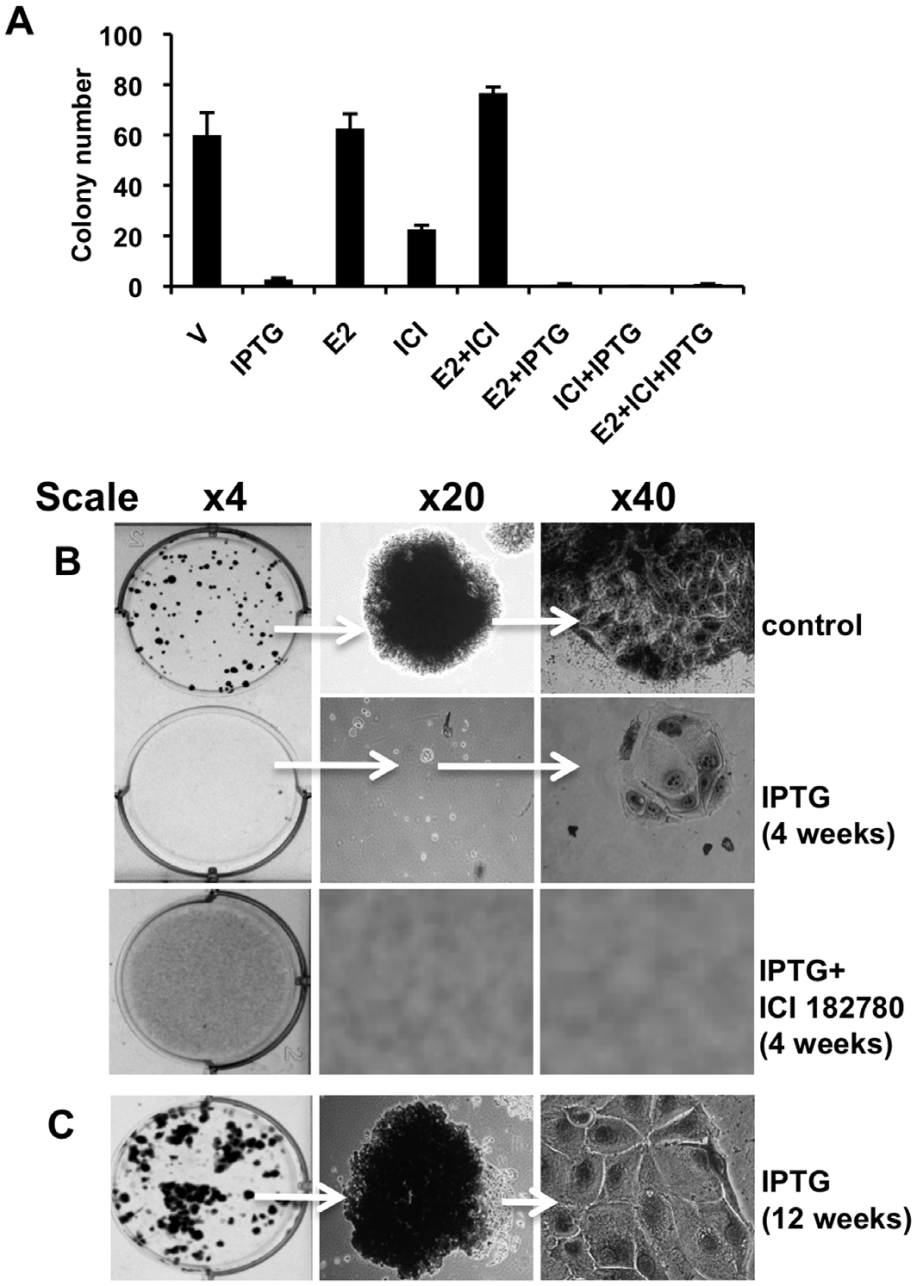
Cell cycle recurrence is abrogated by anti-estrogen ICI 182780. Cells were seeded at 200 cells per well and treated with combinations of IPTG, ICI 182780, E2 and vehicle over a period of 4 and 12 weeks and colonies were visualised with methylene blue staining. **A.** Colonies (+) were counted using a Bio-Rad ChemiDoc XRS + imager in biological triplicate. **B.** Representative wells showing IPTG and ICI 182780 and vehicle (control) treated colonies at week 4. **C.** IPTG treated cells at week 12.

There are at least two potential avenues that MCF-7 cells can use to escape from the constraints of p14ARF-p53 induced senescence based on the literature: cyclin D1 can bind to the estrogen receptor (ER) independent from cdk4/6 and stimulate proliferation [39], [40], [41]; ER can bind to p53 and abrogate cell cycle arrest [42], [43], [44]. Therefore, we explored whether anti-estrogens or estrogens, which bind to ERα and modulate ERα and cyclin D1function, affected p14ARF-latent cell cycle proliferation. To do this we performed clonogenic assays with combinations of ICI 182780, estrogen, and IPTG (Figure 8). At week 4 the number of colonies were measured (Figure 8B). Although treatments with IPTG, ICI 182780, and combination of E2+ IPTG, ICI 182780+ IPTG +E2 reduced colony numbers compared to E2 and vehicle (Figure 8A), only the combination of IPTG and ICI 182780 completely abrogated colony number (Figure 8A and B). The combination of E2+ ICI 182780 enhanced colony number, a phenomenon we consistently observed (Figure 8A). At week 12, colonies were prevalent with IPTG-treatment with noticeable large irregular cells at the periphery (Figure 8B). No colonies formed in the presence of ICI 182780 combined with IPTG (data not shown). Comparison of cyclin D1 protein expression was analysed by Western blot for all the conditions tested and directly compared with p21 and p53 expression (Figure S4). Combination of ICI 182780 and IPTG and ICI 182780 alone reduced the levels of cyclin D1 compared to IPTG alone at 12h and 24h, whereas p21 and p53 protein levels were increased >10 fold and 7 fold respectively under the same conditions (Figure S4). An interesting observation was that downregulation of cyclin D1 was negated in the presence of E2 and ICI 182780 (Figure S4), which in part may explain the enhanced colony numbers we observed with this treatment (Figure 8 A and B).

## Discussion

Understanding the signalling pathways that mediate the different states of cell cycle arrest is fundamental to our understanding of breast cancer development, prevention and better breast cancer treatment. The p14ARF-p53 pathway is well documented as an important defense against cancer by triggering apoptosis/quiescence or senescence [11]. In breast epithelial cancer cells, p14ARF and components of this pathway (p53 and p21) are frequently inactivated and impairs the cell’s ability to control its division and increases their susceptibility to oncogenic signals. In this report we describe the importance of the regulation and localisation of cyclin D1 mediated by p14ARF in both cell cycle inhibition and abnormal proliferation in MCF-7 senescent cells. Induction of p14ARF demonstrated rapid cell cycle arrest (within 6h of induction), and exhibited many of the hallmarks of a senescence phenotype conditional on expression of p14ARF and a functional p53/p21 pathway. We have shown that activation of the p14ARF-p53-p21 pathway both activates Rb through dephosphorylation and inactivates it through degradation. These observations are consistent with its protective role in carcinogenesis. Interestingly, p14ARF promotes accumulation of cyclin D1 at the protein level, but does not directly affect *CCND1* mRNA transcription. Post-translational regulation of cyclin D1 was also confirmed by transcriptional and translational inhibitor treatment, strengthening the quantitative PCR results. In addition, by inhibiting protease activity through addition of MG132 we observe an increase in cyclin D1 protein expression suggesting that cyclin D1 expression was still modified by the ubiquitin-proteasome pathway post-p14ARF expression. These findings raise the question of the importance of cell cycle variations in mRNA levels in regulating protein expression. Although this finding contradicts other reports, where p19ARF (mouse homolog of p14ARF) independently down-regulated cyclin D1 [23], [45], murine and human ARF are quite diverse in their homology especially at the C-terminal [46] and this may in part explain the discrepancies in our findings with those of others [21], [22], [23].

We showed, by systematic knockdown of cyclin D1, p53 and p21, that p21was necessary for the stability of cyclin D1 protein, independent of p53. Suppression of p21, by downregulating p21 or p53, restored the mitogenic effects of cyclin D1 suggesting an epistatic relationship between p14^ARF^/p53/p21 and cyclin D1. These results are consistent with other reports showing, in co-immunoprecipitation experiments, p21 interacts with the cyclin D1/CDK4 complex to inhibit cell proliferation [47], [48], [49] and cyclin D1 requires p21 to maintain protein stability [50]. It has been suggested that p21 protein stabilisation acts by inhibiting cyclin D1 nuclear export thus preventing ubiquitination and degradation [51]. Again our findings are consistent with these reports whereby we showed nuclear retention of cyclin D1 upon p14ARF expression is a prerequisite for rapid cell cycle-inhibition. Others have shown that cyclin D1 is necessary to prevent p21 degradation [38], however cyclin D1 did not regulate p21 at the translational or transcriptional level in our experiments invalidating any suggestion of a feedback mechanism by cyclin D1 in the context of p21 cell cycle control in our model system. Furthermore, repression of p21 had no effect on p53 levels, and cells expressing high p53 protein post-p14ARF induction continued to proliferate. Again this suggests that p21 does not directly feedback and suppress p53 expression in our system (see Table 1 and 2).

**Figure 9:**
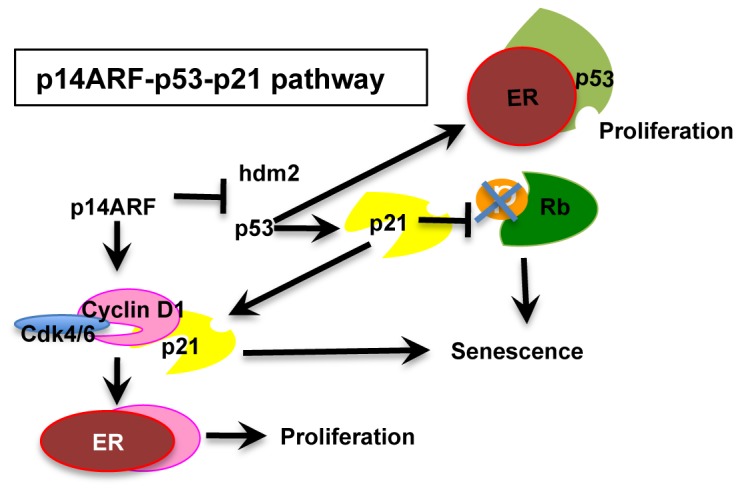
Escape from p14ARF cell cycle arrest (premature senescence in MCF-7 breast cancer cells): A proposed model. P14ARF binds hdm2 and sequesters it to the nucleolus. P53 upregulates p21 and p21 dephosphorylates and downregulates Rb. P21 also binds and inactivates cyclin D1/cdk4/6 complex and induces premature senescence. Two avenues are suggested whereby cells may escape from senescence: ER binds p53 and inactivates p14ARF-p53 inhibitory functions [44]; alternatively ER binds to cyclin D1 with a gain of cell proliferative function [40].

Others have demonstrated that cell cycle recurrence can be mediated by ERα binding to p53 [42], [43], [44], or cyclin D1 [39], [40], [41] in ERα-positive breast cancer cells. In this report anti-estrogen ICI 182780, shown to downregulate ER and cyclin D1, negates latent recurrence in MCF-7 breast cancer cells. Our model, based on our results and those of others, provides two potential escape routes in ER-dependent breast cancer senescent cells: excess nuclear cyclin D1 binds to (1) ERα and/or (2) p53 and potentiates proliferation (Figure 9). These findings may of be of relevance in the development of aged breast cancer biology where late-onset breast cancers are more likely to be ERα positive and p53 wildtype, and loss of wildtype p53 is only seen in a small percentage of aged breast tumours [52].

Senescence is regarded as a strong protective response in aging or abnormal cells, whereby cells can enter irreversible mitogen-refractory growth arrest to prevent tumourigenesis [53], [54], [55], [56]. Although senescent cells are defined as incapable of regeneration, they can accumulate in the tissue, showing marked changes in gene expression patterns and remaining viable with preserved metabolic function [57]. Cells with all the hallmarks of senescence are frequently observed in pre-malignant lesions, but are rare in malignant tumours, suggesting senescence is an early cellular mechanism that prevents carcinogenesis [56]. Conversely, the results of a number of studies also suggest that neoplasia can arise from senescent-like cells [58], [59]. Normal human mammary epithelial cells have been shown to spontaneously escape senescence, as opposed to bypassing senescence, and acquire genomic changes [59].

More recently, neoplastic cells have been shown to spontaneously arise from senescence-associated oxidative DNA damage [58]. Interestingly, it has been shown that after release from p21-induced growth arrest abnormal mitosis endoreplication and development of tumours may occur [60]. The effects of p21 on CDKs are not limited to simple binding and inhibition but may stimulate CDK4/6 complexes depending on its stoichiometry [60]. Our results support an essential role for p21 in the induction of the senescence phenotype. Indeed, further experiments need to be performed to determine whether changes in the stoichiometry of p21/CDK/cyclin D1 allow progression from the senscence phenotype through to endoreplication in these cells. Importantly, these reports and our observations suggest that cancer cells in a senescence-like state are in a dormant, rather than a permanent, state of proliferative arrest and could potentially be a dangerous milieu for tumour relapse. *Accumulation of these abnormal active cells may lead to age-related disease as reviewed by Collado et al, 2005* [61]. It is not unreasonable to suggest that senescence is related to late-onset breast cancer or recurrence of breast cancer post-therapy.

Here we emphasis the impact of post-transcriptional regulation of protein synthesis, protein localisation and microenvironment of cyclin D1, p21 and p53 in regulation of diverse cellular functions such as maintenance of the senescence phenotype or escape and proliferation. Importantly, we have shown that accumulation of nuclear cyclin D1 in latent MCF-7 cells post p14ARF induction is associated with relocalisation of Ki-67 in the nucleus, and loss of genomic integrity in latent MCF-7, eventuating in abnormal nuclear division and aberrant cell division. These results suggest that accelerated senescence through reactivation of the p14–p53 pathway does not entirely remove the threat of cancer and that cyclin D1 plays a promiscuous role in cancer prevention and progression. Although p14ARF rapidly stops cell proliferation, the very nature of cancer cell genomic instability will drive neoplastic formation in senescent cells. As we have shown continuous expression of p14ARF results in the onset of endoreduplication.

This may partly explain the conundrum that high cyclin D1 is associated with non-proliferating breast cells and also aggressive highly proliferative breast cancer. These findings may have clinical significance, in their support of p14ARF and p53 presence in ductal carcinoma in situ and invasive breast cancer and why p14ARF and p53 expression do not prevent tumour growth. This insight into this pervasive pathway towards aneuploidy may reveal cancer vulnerabilities that may be exploited in the clinic. Furthermore these results put a caveat on the use of accelerated senescence as a breast cancer therapeutic strategy and suggest combinational therapies are more effective to reduce ERα breast cancer recurrence. We have shown that ICI 182780 (Faslodex, Fulvestrant), a compound used in the clinic for breast cancer treatment reduces recurrence post p14ARF. We propose that efficacy of breast cancer treatment and reduction of recurrence post therapy where p14ARF-p53 is activated (such as radiation and chemotherapy) can be improved by the use of combination therapies with anti-estrogens in hormone-dependent breast cancer.

## Materials and Methods

### Cell Lines and Culture Conditions

MCF-7 breast cancer epithelial cells (ATCC 30-4500K) and MCF-7p14ARF breast cancer cells [32] and were grown in Dulbecco’s modified Eagle’s medium (Invitrogen) supplemented with 10% foetal bovine serum and insulin (10µg/ml). All cells were cultured in a 37°C incubator with 5% CO_2_. Isopropyl β-D-thiogalactosidase (IPTG) was used to induce p14ARF. All cells were tested mycoplasma free using the mycoAlert Kit (Lonza, Australia).

For experiments investigating the effects of IPTG and hormone treatment, 6-well dishes were seeded at 0.5−1.5×10^5^ cells/well in complete medium unless indicated. Stable clones were seeded 24h prior to induction, and were induced using 5 mM IPTG, 10 nM ICI 182780, 10 nM E2, added directly to the medium and harvested at the times indicated.

### Immunoblot Analysis

Cells were prepared for protein extraction as previously described [62]. Protein was quantified using the BioRad assay (BioRad Lab Inc. CA) and equal amounts of total protein (20µg) were separated by SDS-PAGE and then transferred to PDVF membrane. Proteins were visualised using the ECL detection system (Amersham Pharmacia Biotech, Australia) after incubation with the following primary antibodies: cyclin D1 (DCS-6) (Novacastra Laboratories Ltd, Newcastle-upon-Tyne, UK), anti-phospho-Rb (phosphoSer^780^) (Sigma Aldrich, CA), pRb (G3-245), PharMingen, San Diego, CA), p53 (DO-7, Dako, CA, USA), p21^WAF/CIP1^ (c-19, Santa Cruz), p14ARF (DCS-240, Sigma). Protein abundance was quantified by image analysis using the Kodak image station 4000MM.

### Steroid Preparation

ICI 182780 (Faslodex) [7α-[9-(4,4,5,5,5-pentafluropentyl-sulfinyl)nonyl] and β-estradiol (E2) (Sigma) were prepared in absolute ethanol and stored at −20°C.

### Quantitative Real-time PCR (RT-QPCR)

For gene expression changes, MCF-7 p14^ARF^ cells were seeded at 1.0×10^5^ cells/ml in 10% FCS DMEM for 24 h. Following 12h IPTG (5 mM) (Promega), ICI 182780 (10 nM), or vehicle treatment cells were homogenised in TRIzol (Invitrogen) and RNA extracted according to manufacturer’s instructions.

One µg of total RNA was DNase I treated (Sigma) and then reverse transcribed using the High Capacity cDNA synthesis kit (Applied Biosystems) according to manufacturer’s instructions. Quantitative RT-PCR reactions were performed in a MicroAmp Fast Optical plate (Applied Biosystems) in an ABI PRISM 7900HT Fast Real-Time PCR system (Applied Biosystems). Reactions were prepared in triplicate 20 µl reactions each consisting of 1x TaqMan Fast Gene Expression Master Mix (Applied Biosystems), 1x TaqMan Gene Expression Assay (ACTB, Hs99999903_m1; CDKN1A, hs 00355782_m1; TP53, hs 99999147_ml; CCND1, Hs00277039_m1) (Applied Biosystems), and 10 ng of initial total RNA. Thermocycling consisted of initial hold step at 50°C for 2 min, and 95°C for 10 min followed by 40 cycles of denaturation at 95°C for 15s and annealing and extension at 60°C for 60s with subsequent FAM-dye data acquisition. Relative expression of *CDKN1A*, *TP53* and *CCND1* were normalised to *ACTB* and calculated by the 2^−ΔΔCt^ method [63].

### SiRNA Knockdown

For siRNA knockdown of *CDKN1A*, *TP53* and *CCND1*, cells were seeded at 1.0×10^5^ cells/ml in 10% FCS DMEM for 18 h. siRNA complexes consisted of 18 µl of HiPerFect (Qiagen), 1 µl of 20 nm: hs CDKN1A_6 (Qiagen), hs TP53_9 (Qiagen), CCND1, IDs229 (Applied Biosystems); negative control siRNA, and 100 µl of serum-free DMEM. 1 µl of BLOCK-iT™ Fluorescent Oligo was added to the reaction mix and co-transfected as a RNA transfection. Following 24 h of transfection, cells were stimulated with or without IPTG (5 mM) and protein and RNA extracted.

### Click iT™ EdU Imaging

The EdU (5-ethynyl-2′ -deoxyuridine) is a nucleoside analog of thymidine that is incorporated into DNA only during DNA synthesis allowing the visualisation of newly synthesised DNA [64]. Assays were performed as described previously [32]. Briefly, cells were plated on cover slips in 6-well plates 24h before treatment with 5 mM IPTG. At time intervals indicated cells were treated with 2.5 µM EdU, directly added to the culture medium. Cells were incubated for a further 20h to ensure capture of the majority of proliferating cells. Following EdU addition cells were fixed with 4% paraformaldehyde and permeabilised using acetone for 5 min at −20°C. Incorporation of EdU was observed with Alexa fluor 488 for a further 30 min as described by the manufacturer. Cells were counterstained with Hoechst 33342 (1∶1000) in PBS before mounting on slides using Fluor mount (Sigma) for fluorescent microscopy.

### Immunofluorescence Microscopy and Immunohistochemistry

Cells were seeded at low density on glass cover slips in 6-well plates. Following seeding (24–48h) cells were treated with 5 mM IPTG, 10 nM ICI 182780, vehicle or combinations. At the indicated times cells were fixed with 4% paraformaldehyde for 15 min and permeabilised with acetone for 5 min. Cells were blocked with 2% BSA in PBS for 30 min and incubated with primary antibody for 1h. After washing with PBS cells were incubated with secondary antibody for 1h. During the final washes Hoechst 33342 (Invitrogen) was added 1∶1000 to visualise the nucleus. Antibodies used included: Ki-67 (1∶400, Abcam, Sapphire), p14ARF (1∶300, Zymed-DKSH), p21 and p53 (1∶200 Sigma), Alexus fluor secondary (1∶600, Invitrogen). All images were taken with a Nikon Eclipse Ti-U inverted microscope (Nikon, Tokyo, Japan) with a 5 mega-pixel cooled colour camera and NIS-AR (Advanced research) software. The same magnification (as indicated) was used for comparison. Spot 32 imaging software was used to overlay images, and Image J software used for analysis.

### Statistical Analyses

The relative gene expression differences were analysed by one-way ANOVA with Tukey’s post-test using GraphPad Prism (version 5.01, GraphPad Software, San Diego California USA). A value of P<0.05 was considered significant.

### Clonogenic Assays

Cells were seeded at 200 cells per well in a 6-well plate and treated with combinations of 5 mM IPTG, 10 nM ICI 182780, 10 nM β-estradiol (E2). Medium was changed with additives every 5 days for 4–12 weeks. Colonies were imaged and analysed using a Bio-Rad ChemiDoc XRS + imager (Bio-Rad).

## Supporting Information

Figure S1 **P14ARF rapid exit from early S-phase correlates with a rapid loss of DNA synthesis.** MCF-7p14ARF 4a cells, MCF-7 parent +/− IPTG, were harvested over a time period of 0h-24h and cell cycle phases analysed by flow cytometry. DNA histograms and column graphs: **A.** MCF-7p14ARF IPTG-treated cells; **B.** MCF-7p14ARF 4a cells showing cell cycle distribution. **C.** Column graphs of MCF-7 cells +/− IPTG. S-phase is divided into early (white), mid (black) and late (gray) phases.
(TIF)

Figure S2 **Cell size and granularity correlate with p14^ARF^ expression.** MCF7^p14ARF^ cells were treated with 5 mM IPTG or vehicle for 0-24h. At times indicated cells were washed twice with medium and replaced with fresh medium to remove IPTG. **A.** Western blot: expression of p14ARF was determined at 24h by Western blot. **B.** Cells were incubated for a further 3 days and harvested. Cell cycle distribution was determined using propidium iodide staining and analysed by Flow Cytometry. *Column graph*: percentage of cells in S and G_2_-M-phase of the cell cycle in IPTG and vehicle (PBS) treated cells. The data for each of the experiments represents the mean of three experiments ± SD. **C.** FSC and SSC were used to measure size and granularity.
(TIF)

Figure S3 **Latent effects of p14ARF in MCF-7 cells. A. Individual nucleus within a multinucleated cell has the ability to form a new daughter cell:** Cells were seeded on day 1 and treated with IPTG 24h later. A. At days 15 to 21 photographs were taken of the same multinucleated cell. B. Daughter cells form from individual nuclei; these cells can merge and divide without undergoing conventional cell division. White arrows show nuclei within multinucleated cell undergoing aberrant cell proliferation. Black arrows show spindles joining daughter cells. C. On day 21 cells were trypsinised and photographs taken every few minutes. D. Cells were stained with p14ARF and Ki-67 and show co-localisation within the nucleolus. Ki-67 stained more strongly around the nucleolus, potentially forming a structural ring around the nucleolus. Budding nuclei stain for Ki-67 and p1ARF. BF =  bright phase, Hoechst  =  nucleus.
(TIF)

Figure S4 **Western blot of combinational treatments, IPTG, ICI 182780 and E2 on cyclin D1, p53 and p21 expression.** Cells were treated with combinations of 10 nM ICI 182780, 10 nm E2, 5mM IPTG, and vehicle for 12h and 24h and harvested for protein. Western blot shows comparative analysis of cyclin D1, p53 and p21 protein expression for the treatments as indicated. Experiments were conducted in duplicate independent experiments with similar results. Lanes 1,2,3 and 5 (12h) are also shown as part of Figure 2.
(TIFF)
