# Supplementary figures and images for: p14ARF Post-Transcriptional Regulation of Nuclear Cyclin D1 in MCF-7 Breast Cancer Cells: Discrimination between a Good and Bad Prognosis?
Source: PLoS One. 2012 Jul 30;7(7):e42246. doi: 10.1371/journal.pone.0042246 (PMC3408480; doi:10.1371/journal.pone.0042246)

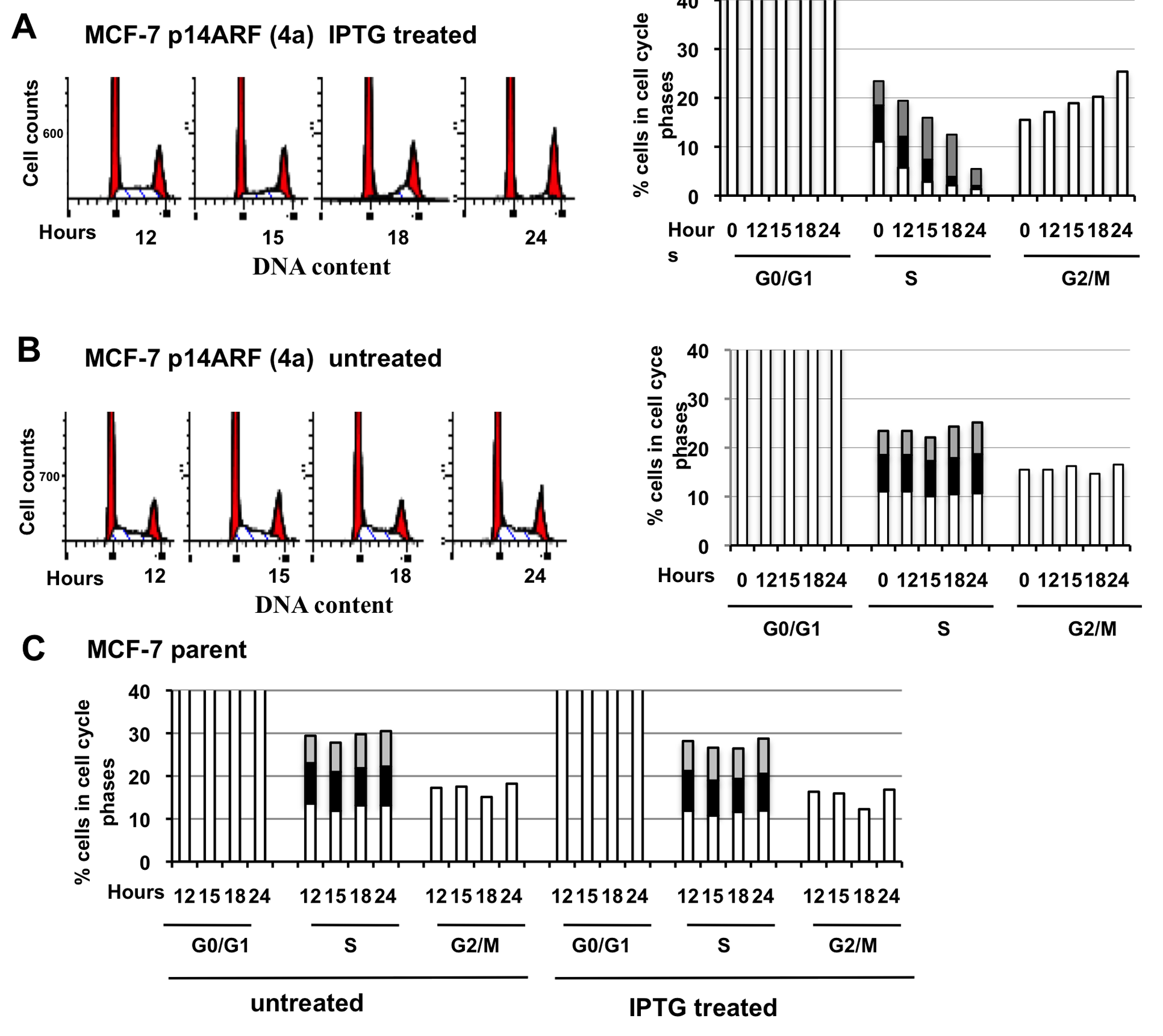

Supplement: Figure S1 — P14ARF rapid exit from early S-phase correlates with a rapid loss of DNA synthesis. MCF-7p14ARF 4a cells, MCF-7 parent +/− IPTG, were harvested over a time period of 0h-24h and cell cycle phases analysed by flow cytometry. DNA histograms and column graphs: A. MCF-7p14ARF IPTG-treated cells; B. MCF-7p14ARF 4a cells showing cell cycle distribution. C. Column graphs of MCF-7 cells +/− IPTG. S-phase is divided into early (white), mid (black) and late (gray) phases. (TIF) [file pone.0042246.s001.tif]

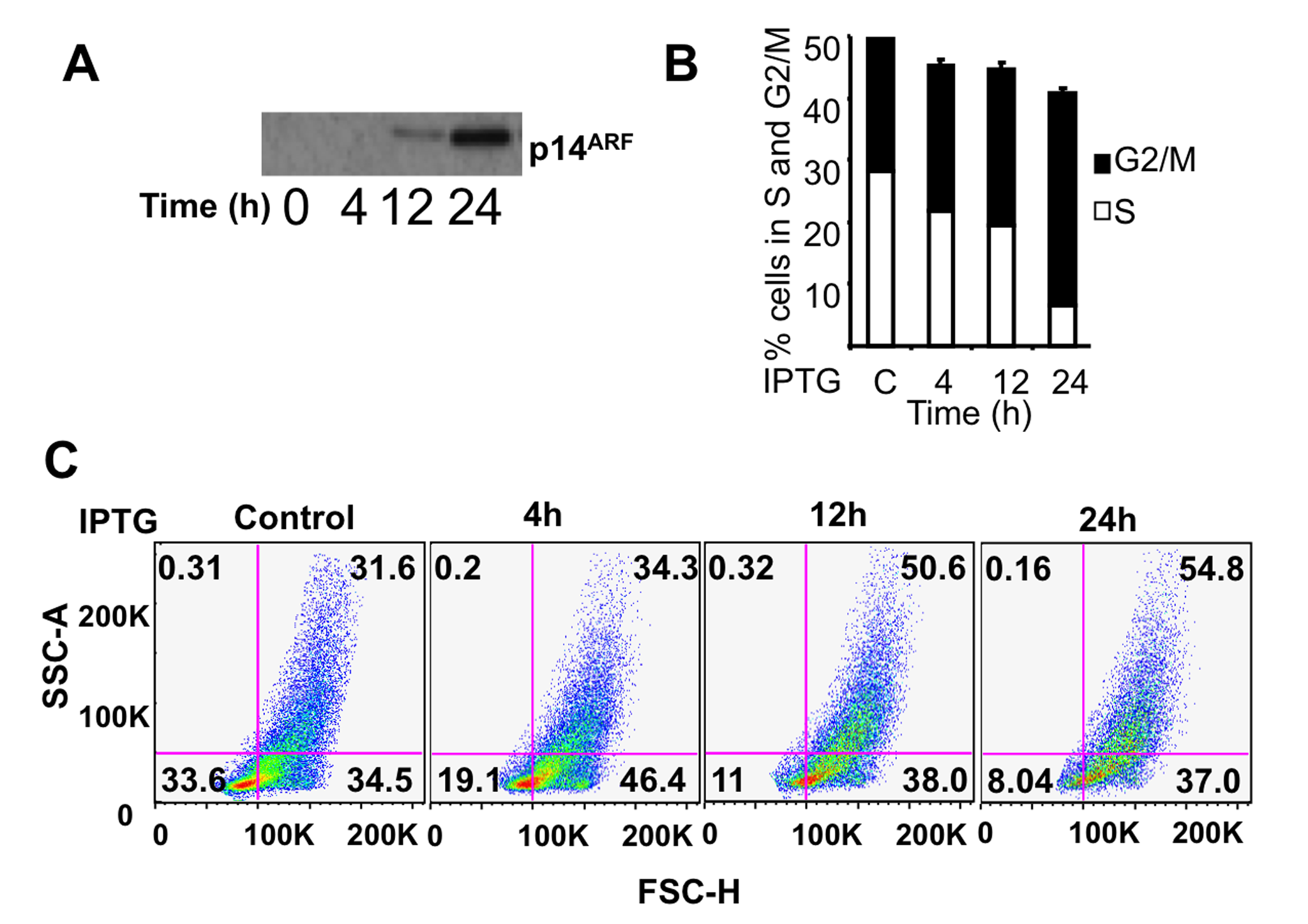

Supplement: Figure S2 — Cell size and granularity correlate with p14ARF expression. MCF7p14ARF cells were treated with 5 mM IPTG or vehicle for 0-24h. At times indicated cells were washed twice with medium and replaced with fresh medium to remove IPTG. A. Western blot: expression of p14ARF was determined at 24h by Western blot. B. Cells were incubated for a further 3 days and harvested. Cell cycle distribution was determined using propidium iodide staining and analysed by Flow Cytometry. Column graph: percentage of cells in S and G2-M-phase of the cell cycle in IPTG and vehicle (PBS) treated cells. The data for each of the experiments represents the mean of three experiments ± SD. C. FSC and SSC were used to measure size and granularity. (TIF) [file pone.0042246.s002.tif]

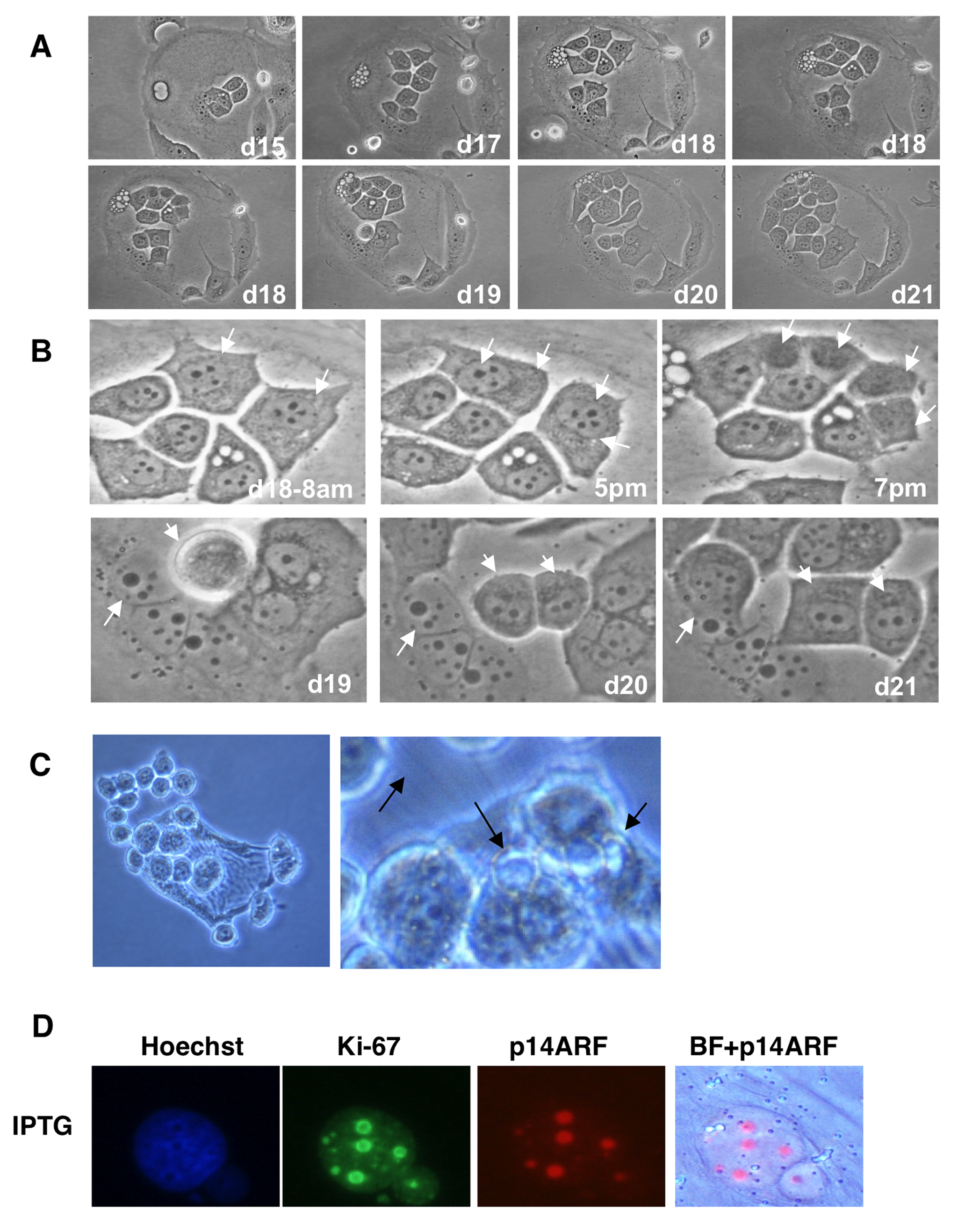

Supplement: Figure S3 — Latent effects of p14ARF in MCF-7 cells. A. Individual nucleus within a multinucleated cell has the ability to form a new daughter cell: Cells were seeded on day 1 and treated with IPTG 24h later. A. At days 15 to 21 photographs were taken of the same multinucleated cell. B. Daughter cells form from individual nuclei; these cells can merge and divide without undergoing conventional cell division. White arrows show nuclei within multinucleated cell undergoing aberrant cell proliferation. Black arrows show spindles joining daughter cells. C. On day 21 cells were trypsinised and photographs taken every few minutes. D. Cells were stained with p14ARF and Ki-67 and show co-localisation within the nucleolus. Ki-67 stained more strongly around the nucleolus, potentially forming a structural ring around the nucleolus. Budding nuclei stain for Ki-67 and p1ARF. BF = bright phase, Hoechst = nucleus. (TIF) [file pone.0042246.s003.tif]

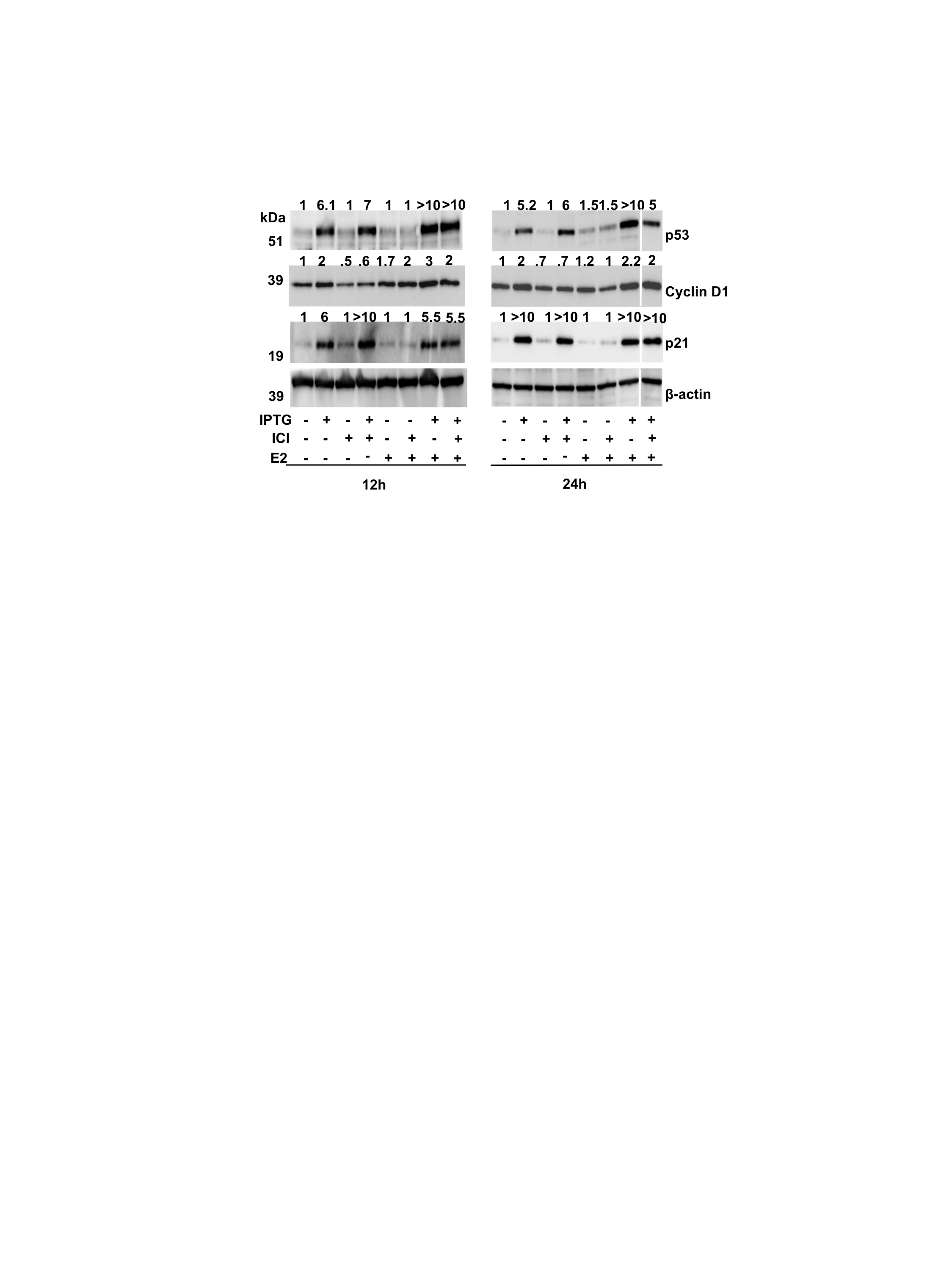

Supplement: Figure S4 — Western blot of combinational treatments, IPTG, ICI 182780 and E2 on cyclin D1, p53 and p21 expression. Cells were treated with combinations of 10 nM ICI 182780, 10 nm E2, 5mM IPTG, and vehicle for 12h and 24h and harvested for protein. Western blot shows comparative analysis of cyclin D1, p53 and p21 protein expression for the treatments as indicated. Experiments were conducted in duplicate independent experiments with similar results. Lanes 1,2,3 and 5 (12h) are also shown as part of Figure 2. (TIFF) [file pone.0042246.s004.tiff]
